# Priorities for multimorbidity management and research in cancer: a Delphi study of Australian cancer survivors, clinicians, and researchers

**DOI:** 10.1007/s11764-024-01686-0

**Published:** 2024-10-02

**Authors:** Rebecca L. Venchiarutti, Haryana Dhillon, Carolyn Ee, Nicolas H. Hart, Michael Jefford, Bogda Koczwara

**Affiliations:** 1 Department of Head and Neck Surgery, Chris O’Brien Lifehouse, Missenden Road, PO Box M5, Camperdown, NSW 2050 Australia; 2 Sydney School of Public Health, Faculty of Medicine and Health, The University of Sydney, Camperdown, NSW Australia; 3 Psycho-Oncology Cooperative Research Group, The University of Sydney, Camperdown, NSW Australia; 4 Western Sydney University, Penrith, NSW Australia; 5 Caring Futures Institute, College of Nursing and Health Sciences, Flinders University, Adelaide, SA Australia; 6 Faculty of Health, Human Performance Research Centre, INSIGHT Research Institute, University of Technology Sydney (UTS), Sydney, NSW Australia; 7 Cancer and Palliative Care Outcomes Centre, Faculty of Health, Queensland University of Technology (QUT), Brisbane, QLD Australia; 8 Exercise Medicine Research Institute, School of Medical and Health Sciences, Edith Cowan University, Perth, WA Australia; 9 Institute for Health Research, The University of Notre Dame Australia, Perth, WA Australia; 10 Department of Health Services Research, Peter MacCallum Cancer Centre, Melbourne, VIC Australia; 11 Australian Cancer Survivorship Centre, Peter MacCallum Cancer Centre, Melbourne, VIC Australia; 12 Sir Peter MacCallum Department of Oncology, University of Melbourne, Melbourne, VIC Australia; 13 College of Medicine and Public Health, Flinders Health and Medical Research Institute, Flinders University, Adelaide, SA Australia; 14 Flinders Centre for Innovation in Cancer, Flinders Medical Centre, Adelaide, SA Australia; 15 Department of Supportive Care and Integrative Oncology, Chris O’Brien Lifehouse, Camperdown, NSW Australia

**Keywords:** Cancer, Comorbidities, Chronic disease, Delphi study

## Abstract

**Purpose:**

Multimorbidity is common in people with cancer and associated with increased complexity of care, symptoms, mortality, and costs. This study aimed to identify priorities for care and research for cancer survivors with multimorbidity.

**Methods:**

A Delphi consensus process was conducted. Elements of care and research were based on Australia’s National Strategic Framework for Chronic Conditions, a literature review, and expert input. In Round 1, health professionals, cancer survivors, and researchers rated the importance of 18 principles, 9 enablers, and 4 objectives. In Round 2, new elements were rated and all elements were ranked.

**Results:**

In Round 1, all elements reached consensus for care delivery; three principles and one enabler did not reach consensus for research and were eliminated. One principle and two enablers were added, reaching consensus. In the final list, 19 principles, 10 enablers, and 4 objectives were included under care delivery; 14 principles, 9 enablers, and 4 objectives were included under research. For care delivery, principles of ‘survivorship’ and ‘self-management’ were ranked highest, and ‘peer support’ and ‘technology’ were the most important enablers. For research, ‘survivorship’ and ‘coordinated care’ were the highest-ranked principles, with ‘peer support’ and ‘education’ the most important enablers.

**Conclusion:**

Most elements apply to the general population and cancer survivors; however, additional elements relevant to survivorship need consideration when managing multimorbidity in cancer survivors.

**Implications for Cancer Survivors:**

Chronic disease frameworks should be more inclusive of issues prioritised by people with, managing, or researching cancer through interdisciplinary approaches including acute and primary care.

## Introduction

Multimorbidity in people with cancer is defined as the presence of at least one other long-term or chronic condition in addition to cancer [1, 2]. It is associated with higher symptom burden, mortality, and health service utilisation [3, 4], and negatively impacts quality of life and survival [5, 6]. Management of multimorbidity is increasingly recognised as a key aspect of comprehensive survivorship care for cancer survivors [7]. In Australia, the *National Strategic Framework for Chronic Conditions* [8] (referred to herein as the *Framework)* lays out a national approach to the prevention and management of chronic conditions and provides guidance for the development and implementation of policies, strategies, actions, and services to address chronic conditions and improve health outcomes.

While the *Framework* includes cancer-specific outcomes as measures of success (e.g. lower cancer incidence and prevalence, probability of dying from cancer, and screening for certain cancers), in general, it is considered disease agnostic. It is unclear whether the *Framework* currently addresses all priorities for the management of multimorbidity in cancer survivors. Further, while the *Framework* moves away from a disease-specific focus, it is possible there are sufficient elements unique to cancer survivors with multimorbidity to justify the revision or generation of a cancer-focused framework for managing multimorbidity. To address this knowledge gap, we aimed to identify a priority set of elements of care delivery and research regarding the management of multimorbidity in cancer survivors in Australia.

## Methods

### Study design and setting

A Delphi consensus process [9] was conducted using a series of surveys to obtain anonymous opinions on elements of care delivery and research from Australian clinicians, researchers, and people with lived experience of multimorbidity and cancer.

### Item generation for Delphi consensus process

Initial items for the Delphi survey were generated from several sources. These included a review of the *Framework* and a scoping review of relevant literature, which involved searches in Google Scholar, reference list checks of relevant publications, and a search of state or national health websites by the first author from December 2022 to March 2023. This process identified 31 elements across three domains: (i) 18 principles (foundational values regarding management of multimorbidity in cancer survivors), (ii) nine enablers (factors required to make optimal management of multimorbidity in cancer survivors possible), and (iii) four objectives (intended focus areas for multimorbidity management in cancer survivors). Ten of the principles were derived from the *Framework* (from eight original statements, two were reworded into separate elements), five from peer-reviewed literature [10–13], and three from health websites [14–16]. All enablers and objectives were derived from the *Framework*, with one enabler and one objective reworded into separate statements as more than two concepts were described within each.

### Delphi consensus method

#### Participants

Participants were purposively sampled based on the primary criterion of specialist knowledge, gained through personal (lived experience) or professional experience of multimorbidity in cancer survivors. Participation was open to health professionals, academic researchers, and cancer survivors with multimorbidity. Cancer survivors were those at any stage along the cancer care continuum who had at least one comorbid condition in addition to cancer. Submission of the questionnaire in REDCap was taken as implied consent. Each survey round was open for 4 weeks.

#### Recruitment

Potential participants were identified through professional networks of the study team, examination of authorship of key literature, and a review of presenters at the Clinical Oncology Society of Australia (COSA) Annual Scientific Meetings from 2020 to 2022. They were invited by direct email from the study coordinator and through promotion by COSA (the peak national body representing cancer health professionals in Australia) via email and social media advertisements which included a link to the online survey. Potential participants were provided an online invitation outlining the study and a link to the online survey with a Participant Information Sheet. Participants were invited to forward the link to colleagues who met the eligibility criteria (passive snowballing).

#### Delphi survey procedures

Prior to completing the survey, interested participants were asked to complete a set of screening questions to verify their expertise, confirm eligibility, and read a Participant Information Sheet. To describe the cohort, the following general information was collected: designation (health professional, academic, or person with lived experience), years in practice and area of specialty (for health professionals only), gender, and state or territory of residence (for cancer survivors) or practice (for health professionals).

The survey was administered electronically using a database hosted by REDCap (Research Electronic Data Capture) [17]. In Round 1, participants were asked to rate the importance of each element (*n* = 31) on a Likert-type scale from 1 (not very important) to 5 (very important) as they pertained to clinical care and research as separate domains (producing a total of 62 ratings). Participants were provided the opportunity to comment on their rating and suggest new elements. Based on the Round 1 results, the questionnaire was revised by the study team to exclude the least important elements and to add new items suggested by participants. In Round 2, participants were asked to firstly rate the importance of each new element on the same Likert scale used in Round 1, and secondly to rank the importance of each element (retained from Round 1 and new elements) as they pertained to both clinical care and research. Due to the large number of principles (*n* = 19) remaining in Round 2 and the potential participant burden, participants were asked to only rank the top 10 principles. A similar approach was taken for enablers and objectives; however, participants were asked to rate all of these elements as the total number was smaller (10 enablers under care delivery, nine enablers under research; and four objectives).

#### Definitions used in the Delphi study

The following operational definitions were provided to participants at the beginning of the survey to provide context for the Delphi study.

1. Multimorbidity: The presence of two or more chronic conditions in a person at the same time. In the context of this study, cancer is considered a chronic condition itself. Therefore, a person with cancer is considered to have multimorbidity if they have another long-term or chronic disease in addition to cancer (e.g. diabetes, cardiovascular disease, pulmonary disease). This may have been diagnosed before (pre-existing) or after cancer was diagnosed.
2. Cancer survivor: Any person who has been diagnosed with cancer at any stage of the treatment and disease trajectory.
3. Importance: The state or fact of being of great significance or value.

### Data analysis

After Round 1, elements with a mean score ≥ 4 out of 5 (indicating participants rated these elements as highly important) and for which ≥ 70% of respondents rated the element as ‘4’ or ‘5’ (reflecting consensus) were retained for the Round 2 survey. Elements that did not meet both thresholds were discussed among the study team, who came to a consensus about the retention or removal of the element for Round 2. Qualitative (written feedback in open-ended comment boxes) data provided by participants were considered during this process. The same principles of analysis were used when analysing Round 2 results to produce the final list of elements.

Results of the surveys are presented using the following summary statistics: mean score (higher score indicates greater importance), standard deviation (lower standard deviation indicates greater consensus), and percentage of respondents rating the element as ‘4’ or ‘5’ (higher percentages representing greater agreement and a proportion of ≥ 70% reflecting consensus). Rankings were determined by summing the ranking scores where a higher score represented a higher rank (most important). Only surveys with complete responses were analysed.

### Ethics approval

The study was approved by the Sydney Local Health District (RPA Zone) Human Research Ethics Committee (Protocol No. X23-0209 & 2023/ETH01019).

## Results

### Participant characteristics

**Fig. 1:**
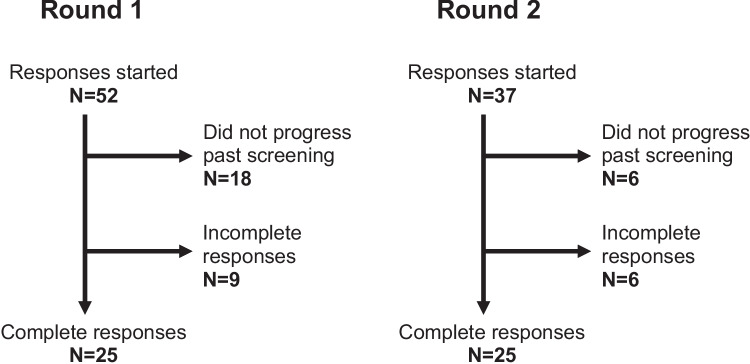
Flow of participants through the two rounds of the Delphi study

In both rounds, 25 complete responses were provided (Fig. 1). There was representation from each of the three targeted groups (Table 1).

**Table 1:** Participant characteristics

Characteristic	Round 1 (*n* = 25) *N* (%)	Round 2 (*n* = 25) *N* (%)
Gender		
Female	21 (84)	16 (64)
Male	4 (16)	9 (36)
Role		
Cancer survivors*	8 (32)	4 (16)
Researcher/academic	3 (12)	4 (16)
Health professional	14 (56)	17 (68)
*General practitioner*	2 (8)	1 (4)
*Nurse*	3 (12)	3 (12)
*Cancer care coordinator*	2 (8)	-
*Oncologist*	2 (8)	5 (20)
*Other^*	5 (20)	7 (28)
Location		
New South Wales	13 (52)	9 (36)
Queensland	5 (20)	2 (8)
Victoria	5 (20)	8 (32)
South Australia	1 (4)	-
Western Australia	1 (4)	1 (4)
Not stated	-	5 (20)
Years in practice#		
< 5 years	1 (4)	2 (8)
5–10 years	1 (4)	3 (12)
10–20 years	6 (24)	4 (16)
> 20 years	9 (36)	7 (28)
Not stated	-	5 (20)

*People with self-reported lived experience of cancer and multimorbidity

^Occupational therapist, psychologist, cardiologist, exercise physiologist, integrative oncology naturopath, acupuncturist

^#^Clinicians and researchers/academics only

### Importance ratings of elements

In Round 1, all principles, enablers, and objectives were rated as important (mean score ≥ 4) and reached consensus for care delivery (Table 2). However, for research, three principles (‘accountable care’, ‘transparent care’, and ‘shared responsibility’) and one enabler (‘clinical governance’) did not reach consensus and were not included in Round 2’s prioritisation for research. All four objectives reached consensus when considered in relation to research.

**Table 2:** Importance ratings of elements included in Delphi survey Round 1

Element	Care delivery		Research	
	Mean score (SD)	Consensus (%)	Mean score (SD)	Consensus (%)
Principles				
Equity: individuals with comorbid chronic diseases and cancer receive safe, high-quality health care irrespective of background or personal circumstance	4.92 (0.28)	100%	4.28 (0.94)	88%
Evidence-based: rigorous, relevant, and current evidence informs best practice and strengthens the knowledge base to effectively prevent and manage chronic conditions	4.64 (0.57)	96%	4.60 (0.71)	88%
Access: high standard, appropriate support, and services available, accessible, equitable, and affordable	4.84 (0.37)	100%	4.28 (0.98)	84%
Safety: prevention and reduction of risks, errors, and harms that occur to patients during provision of health care	4.56 (0.65)	92%	4.24 (0.83)	76%
Person-centred approaches: the health system is shaped to recognise and value the needs of individuals, their careers, and their families, to provide holistic care and support	4.60 (0.50)	100%	4.48 (0.71)	88%
Whole-person care: care that is patient-centred and considers the optimal physical, behavioural, emotional, and social wellness and outcomes of every individual	4.84 (0.37)	100%	4.56 (0.58)	96%
Value-based care: delivery of care in a manner that improves health outcomes that matter to patients, experiences of giving and receiving care, and that improve effectiveness and efficiency of care	4.60 (0.50)	100%	4.32 (0.85)	96%
Sustainability: strategic planning and responsible management of resources deliver long-term improved health outcomes	4.36 (0.76)	92%	4.28 (0.98)	84%
Accountable care: responsibilities are clear and accountable and achieve the best value with public resources	4.32 (0.69)	88%	4.04 (1.06)	68%
Transparent care: decisions are clear and achieve the best value with public resources	4.16 (0.85)	80%	3.84 (1.07)	56%
Collaboration: identify and act upon opportunities for care providers to cooperate and collaborate	4.64 (0.49)	100%	4.40 (0.91)	92%
Partnerships: identify and act upon opportunities for care providers to partner with individuals with cancer and comorbid conditions and groups that represent them	4.52 (0.59)	96%	4.28 (1.06)	84%
Shared responsibility: all parties understand, accept, and fulfil their roles and responsibilities, to ensure enhanced health outcomes for all Australians	4.24 (0.60)	92%	3.96 (1.10)	68%
Care integration: coordination within and between different parts of the health system and service providers to better meet the needs of people with chronic conditions	4.84 (0.37)	100%	4.68 (0.48)	100%
Coordinated care: providing care that is consistent, holistic, and coordinated across the health system to manage their chronic conditions	4.64 (0.49)	100%	4.72 (0.46)	100%
Care navigation: support for coordinated, person-centred care to help individuals navigate the health system	4.60 (0.58)	96%	4.48 (0.59)	96%
Survivorship: focus on the health and well-being of an individual with cancer, encompassing physical, mental, emotional, social, and financial impacts of cancer	4.72 (0.54)	96%	4.68 (0.48)	100%
Self-management: the skills and ability of an individual to undertake activities to live well with one or more chronic conditions	4.56 (0.51)	100%	4.44 (0.65)	92%
Respectful care: care that has regard for the feelings, wishes, and rights of individuals to be treated with dignity, care, and compassion	4.88 (0.44)	100%	3.96 (0.73)	72%
Enablers				
Clinical governance: relationships and responsibilities established by a health organisation between key stakeholders (state/territory department of health, governing body, executive, workforce, patients, consumers, other stakeholders) to deliver good outcomes	4.40 (0.58)	96%	3.84 (1.14)	64%
Leadership: supports evidence-based shared decision-making and encourages collaboration to enhance health system performance	4.40 (0.76)	84%	4.08 (1.04)	72%
Skilled cancer care workforce: a suitably trained, resourced, and distributed skilled cancer care workforce is supported to work to its full scope of practice and is responsive to change	4.68 (0.48)	100%	4.44 (0.77)	92%
Skilled non-cancer care workforce: a suitably trained, resourced, and distributed skilled non-cancer workforce that can work with and support the cancer care workforce	4.20 (0.71)	84%	3.96 (1.05)	72%
Health literacy: people are supported to understand information about health and health care, to apply that information to their lives to use it to make decisions and take actions relating to their health	4.48 (0.59)	96%	4.28 (0.74)	84%
Research and quality improvement: quality health research accompanied by the translation of research into practice and knowledge exchange strengthens the evidence base and improves health outcomes	4.48 (0.65)	92%	4.72 (0.54)	96%
Data and information: the use of consistent quality data and real-time data sharing to enable monitoring, inform practice, and quality improvement to achieve better health outcomes	4.36 (0.70)	88%	4.36 (0.64)	92%
Technology: supports more effective and accessible prevention and management strategies and offers avenues for new and improved technologically driven initiatives	4.20 (0.65)	88%	4.24 (0.88)	80%
Resources: adequate allocation, appropriate distribution, and efficient use of resources, including funding and reimbursement for care delivery, to address identified health needs over the long term	4.52 (0.65)	92%	4.16 (1.03)	76%
Education: information is provided to patients aimed at improving knowledge and skills needed to promote health	4.76 (0.44)	100%	4.28 (0.79)	80%
Peer support: individuals are made aware of opportunities to connect with people with similar health conditions and experiences managing their health	4.20 (0.71)	84%	3.92 (0.57)	80%
Objectives				
Prevention: focus on prevention of chronic disease through health promotion and risk reduction for people with cancer	4.52 (0.71)	96%	4.32 (0.85)	84%
Quality of life: efficient, effective, and appropriate care to support people with cancer living with chronic conditions to *optimise quality of life*	4.72 (0.46)	100%	4.68 (0.48)	100%
Prolonged survival: efficient, effective, and appropriate care to support people with cancer living with chronic conditions to *prolong survival*	4.16 (0.85)	80%	4.20 (0.65)	88%
Priority populations: targeted action for priority populations* to ensure access to quality, safe health care, and relevant information by providing - Culturally safe and appropriate services - Accessible health services that are effective, high-quality, and affordable; and - Flexible service options	4.44 (0.71)	88%	4.40 (0.87)	84%

*Priority populations include, but are not limited to the following: Aboriginal and Torres Strait Islander people, people from culturally and linguistically diverse backgrounds, older Australians, carers of people with chronic conditions, people experiencing socio-economic disadvantage, people living in remote or rural and regional locations, people with disability, people with mental illness, people who are or have been incarcerated, children, teenagers, and young adults, LGBTQI + Australians, refugees

Based on participant feedback, three new elements were introduced into the list for importance rating and ranking in Round 2—one principle (‘respectful care’) and two enablers (‘education’ and ‘peer support’) and wording for one principle and three enablers were modified. Each new element reached consensus in Round 2. Due to an overlap in concepts, two enablers (‘skilled cancer workforce’ and ‘skilled non-cancer workforce’) were combined under one element (‘health workforce’) with wording modifications to reflect the inclusion of both the cancer and non-cancer workforce. Based on participant suggestions, one additional population (people living in isolation) was added to the list of priority populations under Objective 4.

### Prioritisation rankings of final list of elements

In Round 2, participants ranked each element from most important to least important within the categories of principles, enablers, and objectives. Rankings of the final list of elements are shown in Table 3. Within principles, ‘survivorship’ was ranked as the most important under both care delivery and research. ‘Self-management’ and ‘equity’ were ranked second and third most important, respectively, under care delivery, but only seventh and ninth, respectively, under research. Under research, ‘coordinated care’ and ‘person-centred approaches’ were ranked second and third most important by participants. Of the top 10 ranked principles, two did not make the top 10 ranking under research (‘sustainability’ and ‘value-based care’).

**Table 3:** Elements of multimorbidity care delivery and research ranked in descending order of priority (Delphi survey Round 2)

Element	Care delivery		Research	
	Score	Rank	Score	Rank
Principles				
Survivorship: focus on the health and well-being of an individual with cancer, encompassing physical, mental, emotional, social, and financial impacts of cancer	117	1	142	1
Self-management: the skills and ability of an individual to undertake activities to live well with one or more chronic conditions	107	2	86	7
Equity: individuals with comorbid chronic diseases and cancer receive safe, high-quality health care irrespective of background or personal circumstance	102	3	81	9
Access: high standard, appropriate support, and services available, accessible, equitable, and affordable	92	4	106	5
Collaboration: identify and act upon opportunities for care providers to cooperate and collaborate	88	5	82	8
Care integration: coordination within and between different parts of the health system and service providers to better meet the needs of people with chronic conditions	87	6	107	4
Coordinated care: providing care that is consistent, holistic, and coordinated across the health system to manage their chronic conditions	83	7	115	2
Person-centred approaches: the health system is shaped to recognise and value the needs of individuals, their careers, and their families, to provide holistic care and support	82	8 (equal)	110	3
Sustainability: strategic planning and responsible management of resources deliver long-term improved health outcomes	82	8 (equal)	58	15
Value-based care: delivery of care in a manner that improves health outcomes that matter to patients, experiences of giving and receiving care, and that improve effectiveness and efficiency of care	81	10	63	13
Whole-person care: care that is patient-centred and considers the optimal physical, behavioural, emotional, and social wellness and outcomes of every individual	80	11	94	6
Evidence-based: rigorous, relevant, and current evidence informs best practice and strengthens the knowledge base to effectively prevent and manage chronic conditions	68	12	68	12
Care navigation: support for coordinated, person-centred care to help individuals navigate the health system	67	13	77	10
Respectful care—care that has regard for the feelings, wishes, and rights of individuals to be treated with dignity, care and compassion	51	14	37	16
Safety: prevention and reduction of risks, errors, and harms that occur to patients during provision of health care	49	15	70	11
Partnerships: identify and act upon opportunities for care providers to partner with individuals with cancer and comorbid conditions and groups that represent them	45	16	59	14
Transparent care: decisions are clear and achieve the best value with public resources	26	17	N/A	N/A
Accountable care: responsibilities are clear and accountable and achieve the best value with public resources	25	18	N/A	N/A
Shared responsibility: all parties understand, accept and fulfil their roles and responsibilities, to ensure enhanced health outcomes for all Australians	23	19	N/A	N/A
Enablers				
Peer support: individuals are made aware of opportunities to connect with people with similar health conditions and experiences managing their health	197	1	179	1
Technology: supports more effective and accessible prevention and management strategies and offers avenues for new and improved technologically driven initiatives	176	2	123	5
Data and information: the use of consistent quality data and real-time data sharing to enable monitoring, inform practice, and quality improvement to achieve better health outcomes	159	3	102	7
Education: information is provided to patients aimed at improving knowledge and skills needed to promote health	153	4	145	2 (equal)
Health literacy: people are supported to understand information about health and health care, to apply that information to their lives to use it to make decisions and take actions relating to their health	151	5	124	4
Leadership: supports evidence-based shared decision-making and encourages collaboration to enhance health system performance	130	6	145	2 (equal)
Research and quality improvement: quality health research accompanied by the translation of research into practice and knowledge exchange strengthens the evidence base and improves health outcomes	135	7	98	8
Clinical governance: relationships and responsibilities established by a health organisation between key stakeholders (state/territory department of health, governing body, executive, workforce, patients, consumers, other stakeholders) to deliver good outcomes	108	8	N/A	N/A
Resources: adequate allocation, appropriate distribution, and efficient use of resources, including funding and reimbursement for care delivery, to address identified health needs over the long term	105	9	118	6
Skilled health workforce: a suitably trained, resourced, and distributed skilled cancer care workforce is supported to work to its full scope of practice and is responsive to change	68	10	82	9
Objectives				
Prolonged survival: efficient, effective, and appropriate care to support people with cancer living with chronic conditions to *prolong survival*	82	1	85	1
Priority populations: targeted action for priority populations* to ensure access to quality, safe health care, and relevant information by providing - Culturally safe and appropriate services - Accessible health services that are effective, high-quality, and affordable; and - Flexible service options	70	2	62	2
Prevention: focus on prevention of chronic disease through health promotion and risk reduction for people with cancer	50	3	50	4
Quality of life: efficient, effective, and appropriate care to support people with cancer living with chronic conditions to *optimise quality of life*	48	4	53	3

N/A = did not reach consensus for inclusion under ‘Research’ and were not included in the ranking exercise

*Priority populations include, but are not limited to the following: Aboriginal and Torres Strait Islander people, people from culturally and linguistically diverse backgrounds, older Australians, carers of people with chronic conditions, people experiencing socio-economic disadvantage, people living in remote or rural and regional locations, people with disability, people with mental illness, people who are or have been incarcerated, children, teenagers, and young adults, LGBTQI + Australians, refugees, people in isolation

**Fig. 2:**
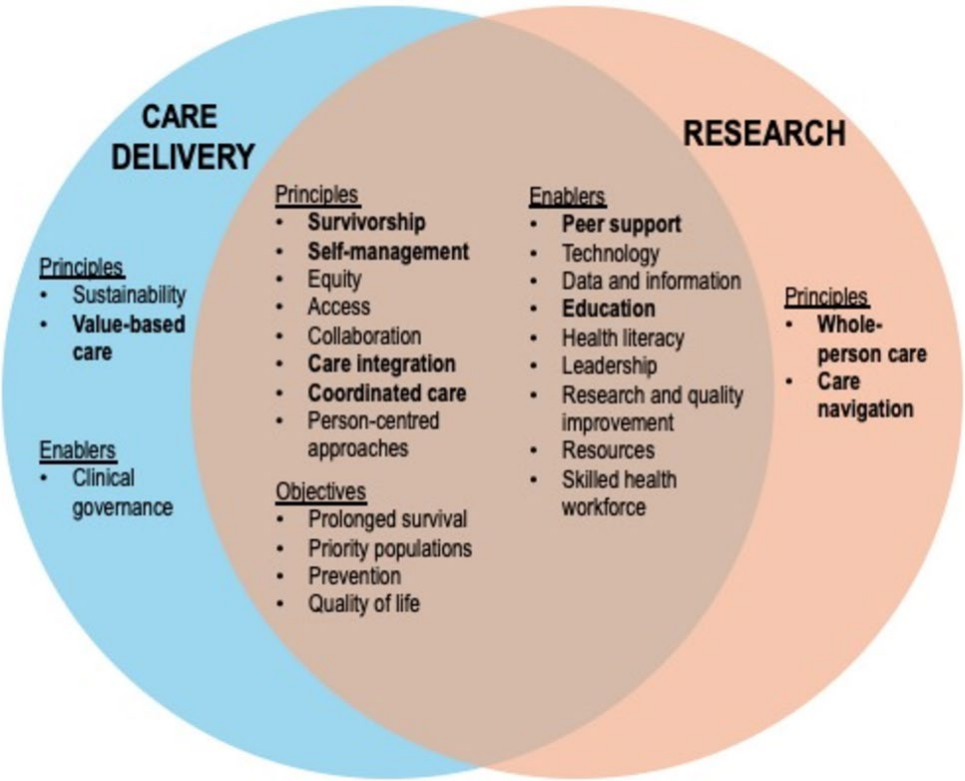
Priority elements for multimorbidity management in people with cancer for care delivery (blue) and research (orange). Common elements are included in the centre, with highly ranked elements unique to either care delivery or research in the outer areas on the left and right respectively. New elements not previously identified in the *National Strategic Framework for Chronic Conditions* are bolded

Within the enablers, ‘peer support’ was ranked as the most important under both care delivery and research. ‘Technology’ and ‘data and information’ were ranked second and third under care delivery. Under research, ‘education’ and ‘leadership’ were tied for the second most important enablers. Within objectives, ‘prolonged survival’ and ‘priority populations’ both ranked first and second in terms of importance under both care delivery and research. Figure 2 presents a graphical framework of the most highly ranked elements of multimorbidity care delivery and research.

## Discussion

This study aimed to identify priorities for multimorbidity care delivery and research in cancer survivors. The findings indicated many elements within an existing chronic disease management framework [8] were applicable to cancer survivors with multimorbidity. However, there were additional items highly prioritised including principles of ‘survivorship’ and ‘self-management’ and enablers of ‘peer support’ and ‘education’. The highest ranked principle, for both care delivery and research domains, was ‘survivorship’, which recognises this critical post-treatment phase for cancer survivors [18].

Self-management and a focus on quality of life were also highly ranked. These concepts are recognised as essential elements of the effective management of multimorbidity in the general population [19], and the high rankings of these elements with regard to multimorbidity suggest consistency between cancer and non-cancer settings. Likewise, among enablers, ‘peer support’ was the highest-ranked element among both care delivery and research. Peer support, in which individuals are made aware of opportunities to connect with other people with similar health conditions and experiences, was an element added after Round 1. Peer support is perceived as acceptable in addressing unmet needs among people with cancer [20], but uptake in practice has been limited. The effectiveness of peer support in multimorbidity is unclear, owing in part to a lack of consistency in definitions and conduct and reporting of research [21] and possibly an overall lack of research being conducted in this area. Many peer support interventions include education, self-management, discussion with peers, and reciprocal support in either group or one-on-one settings delivered by telephone, in-person, or online [21]; however, most studies report positive but non-significant effects and there is a lack of consistency in how peers are defined. However, that it was rated highly by participants, but not evident in the initial literature review, suggests it is a significant gap in care delivery and research and for which additional focus is warranted. Education is often included in interventions to improve outcomes for people with chronic conditions [22] and is often delivered in combination with other elements such as self-management [23]. This highlights that attempts to operationalise care delivery priorities should not just address elements in isolation but consider how they interact and may be delivered to complement each other.

Two principles—‘whole-person care’ and ‘care navigation’—were elements prioritised in the top 10 for research, but not for care delivery. With regard to ‘whole-person care’, this could reflect recent calls for further research seeking to understand how this comprehensive and holistic approach to survivorship could be delivered in practice, and the economic viability of these approaches, especially in the primary care setting [24]. This would be essential to design optimal models of care for individuals with multimorbidity. ‘Care navigation’ has been a recent focus in Australia with a commitment in the Australian Cancer Plan to establish a cancer navigation service under the new Australian Cancer Nursing and Navigation Program [25]. This announcement coincided with Round 2 of this Delphi study, which sought prioritisation of elements, and could explain why ‘care navigation’ was more highly prioritised for research rather than care delivery. Research to ensure equitable access to and delivery of this care navigation service is essential, especially for priority populations such as those with socioeconomic disadvantage, First Nations Australians, and culturally and linguistically diverse persons who are at greater risk of multimorbidity [26–28] and experience more challenges engaging with the health system [29].

Multimorbidity requires coordination and alignment of multiple services that extend beyond focusing on a single disease or body system, to encompass multi- and interdisciplinary care crossing primary, secondary, and tertiary care [30]. In the context of cancer, this care should be delivered across the care continuum from prevention and diagnosis, treatment, survivorship, and end-of-life care. These philosophies are echoed in the final priority list of elements of care delivery we identified, with principles of ‘access’, ‘care integration’, ‘sustainability’, and ‘coordinated care’ among the top ten priorities in this domain. Additionally, a person-centered approach is imperative given multimorbidity is not a single heterogeneous disease or condition. Typically, approaches to addressing the care of people with multimorbidity in the acute setting have focused on managing individual diseases (e.g. cancer, chronic obstructive pulmonary disease, diabetes) as separate entities. This is in contrast to primary care, where the focus is more frequently on the whole person rather than individual disease. These strengths of primary care can be used to promote the priorities identified in our study, i.e. whole-person care and person-centred approaches across the health system for people with cancer. Primary care teams are well placed to manage the complexity of multimorbidity. In addition, greater recognition of cancer as a comorbid illness by GPs and promotion of chronic disease management plans for people with cancer after discharge from active treatment could improve the coordination of care for people with cancer and multimorbidity by improving access to other health services such as allied health. However, it is critical that there is adequate funding for primary care systems, sufficient availability of primary care providers, and flexible healthcare delivery models, as strong primary care systems reduce health inequities and healthcare costs, reliance on acute care, and improve overall patient outcomes [31, 32]. Building the health workforce was identified as an enabler of optimal multimorbidity care, and there should be an emphasis on building capacity in primary care to care for individuals with multimorbidity given the existing skills and expertise in the workforce.

A strength of our study is that we purposively sampled clinicians, researchers, and people with a lived experience of cancer and multimorbidity to elucidate a diverse range of views for a single, unifying framework. Research has shown evidence of low levels of agreement between health outcomes and treatment priorities of patients with multimorbidity and clinicians [33], so our approach sought to include representation from all potential stakeholders (clinicians, researchers, and cancer survivors) to whom the framework might apply. Our work has identified important priorities for multimorbidity and cancer.

The next step is to determine how the findings can be operationalised in clinical practice and research, to ensure critical aspects of cancer care and multimorbidity are addressed in the real world. With regard to priorities for care delivery, for elements focused on individuals such as ‘self-management’, ‘care-integration’, and ‘coordinated care’, inclusion in survivorship care plans is recommended, though it is noted uptake of survivorship care plans is low in practice despite being viewed favourably by survivors and providers [34]. There could be greater involvement by primary care, including in multimorbidity multidisciplinary team meetings [35] as well as monitoring outcomes of multimorbidity management more systematically. The core elements identified in this study span multiple settings in the health system, from primary care to acute hospitals, post-treatment survivorship, and end-of-life care. Consequently, interdisciplinary approaches for managing multimorbidity in people with cancer are essential.

### Limitations

Despite our attempts, some key groups involved in managing comorbid illness did not participate in this Delphi study, such as cardiologists, endocrinologists, pain specialists, pharmacists, and palliative care clinicians. It is possible our small sample lacks sufficient diversity, particularly among priority groups who are at greater risk of multimorbidity. A limitation of our study is that the rating and ranking processes are inherently subjective [36]. It is possible, with our relatively small sample, that important items related to multimorbidity in cancer may have been missed. Lastly, this study provides an Australian perspective, which will likely impact replicability and generalisability in other settings as priorities may differ depending on different healthcare resources and models.

## Conclusion

Many elements of multimorbidity management for cancer survivors overlap with pre-existing general chronic disease frameworks. While there are sufficient nuances for people with cancer, given a move away from disease-focused frameworks, chronic disease frameworks could aim to be more inclusive of the issues prioritised by people with, managing, or researching cancer rather than the creation of a distinct framework. Operationalising elements of care delivery should consist of interdisciplinary approaches including acute and primary care.

## Data Availability

The de-identified data we analysed are not publicly available, but requests to the corresponding author for the data will be considered on a case-by-case basis.
